# Editor's Letter-Box

**Published:** 1889-02-23

**Authors:** George Thurlow

**Affiliations:** Hon. Secretary


SIR,—My attention has only recently been called to a paragraph in your issue of the 5th ult., stating that the managers of the All Saints' Convalescent Hospital, Eastbourne, have gone the length of refusing admittance to *Truth.* I beg to assure you the statement is not correct; the managers never did anything of the kind. They have no thought or intention of not allowing *Truth*, which is constantly being sent to the patients, and is frequently bought by the patients themselves.

GEORGE THURLOW, Hon. Secretary.

